# Upadacitinib for the Management of Alopecia Totalis and Subtotalis in Pediatric Patients: A Case Series

**DOI:** 10.3390/reports9010073

**Published:** 2026-02-28

**Authors:** Waleed Alajroush, Huda Alrwebah, Abdulelah Alghamdi, Salam Alanazi, Saif Alagha, Sawsan Alharthi

**Affiliations:** 1 Department of Pediatric Dermatology, King Abdullah Specialized Children’s Hospital, Riyadh 11426, Saudi Arabia; 2 Division of Dermatology, Department of Medicine, King Abdulaziz Medical City, Riyadh 11426, Saudi Arabia; 3 College of Medicine, King Saud bin Abdulaziz University for Health Sciences, Riyadh 11481, Saudi Arabia

**Keywords:** alopecia areata, JAK inhibitor, upadacitinib, pediatric population

## Abstract

**Background**: Alopecia areata is an autoimmune disorder characterized by nonscarring hair loss, which can progress to alopecia totalis or universalis. While JAK inhibitors have shown efficacy in severe cases, evidence in pediatric and adolescent populations remains limited. This study evaluated the efficacy and safety of upadacitinib in pediatric patients with alopecia totalis and subtotalis. **Methods**: This is a retrospective case series that included eight patients aged 9 to 14 years treated with upadacitinib 15 mg daily and followed for up to two years. Clinical response, treatment duration, adverse effects, and laboratory results were monitored. **Results:** All patients demonstrated clinical improvement, with most achieving complete or near-complete regrowth of scalp, eyebrow, and eyelash hair. The median regrowth time was 3 months. Mild acne was observed in two patients; no serious side effects occurred. **Conclusions**: In this case series, upadacitinib was associated with encouraging clinical responses and was well-tolerated by most of our patients; however, larger-scale studies are needed to confirm its efficacy and long-term safety.

## 1. Introduction

Alopecia areata (AA) is an autoimmune inflammatory dermatological disease targeting anagen hair follicles, leading to discrete, smooth patches of nonscarring hair loss frequently involving the scalp [1]. Other affected hair-bearing areas include the eyebrows, eyelashes, and beard. In extreme instances, the entire scalp (alopecia totalis) or body (alopecia universalis) may be impacted [1,2].

AA involves immune dysregulation and loss of immune privilege of hair follicles. This leads to reduced immunosuppressive signals such as tumor growth factor-beta and alpha-melanocytic stimulating hormone and increased major histocompatibility complex class I expression, activating natural killer cells. These cells release interferon-gamma and interleukin (IL)-15, triggering inflammation through the Janus kinase (JAK)–signal transducer and activator of transcription (JAK-STAT) pathway. This pathway is a key target of JAK inhibitors, which have shown promise in treating AA and other inflammatory skin diseases [3].

Multiple local and systemic therapies have been used for AA, but most clinical trials focus on adults, with limited data in children. In pediatric AA, high-potency topical corticosteroids are the first-line treatment. Although intralesional corticosteroids are effective, their use in children is limited due to pain and lack of extensive safety data [4].

Among the systemic therapies for AA, JAK inhibitors such as the Food and Drug Administration (FDA)-approved baricitinib have shown promising efficacy and safety, particularly in adults [5]. Tofacitinib and ruxolitinib have been used in pediatric and adolescent patients, with some of them achieving excellent results [6].

We aim to highlight the clinical outcomes of upadacitinib in pediatric patients with AA through this retrospective case series conducted at King Abdullah Specialist Children’s Hospital in Riyadh. Eight patients aged 9–14 received upadacitinib 15 mg daily and were followed for two years. Data on demographics, disease history, prior treatments, clinical response, and safety were collected from electronic records. Effectiveness was assessed by measuring hair regrowth, and safety was monitored through reported adverse events and lab evaluations. Follow-up was conducted monthly after initiation, and then every 3–6 months.

## 2. Methods

This is a retrospective case series conducted at King Abdullah Specialized Children’s Hospital. The inclusion criteria were a diagnosis of alopecia areata of any subtype and being treated with upadacitinib. Eight patients aged 9 to 14 were included in this study. Data were extracted from the electronic medical records of the patients and contained both prospective and retrospective elements. The collected variables included demographics, disease subtype, disease duration, disease extent, prior treatments, follow-up duration, and adverse events. All eight patients underwent baseline evaluation prior to upadacitinib initiation and routine testing throughout the treatment duration. Baseline evaluation included complete blood counts, liver function tests, lipid profile, and screening for tuberculosis and hepatitis B and C. A complete blood count, liver function test, and lipid profile were repeated at each follow-up visit every 3–4 months. Tuberculosis and hepatitis B and C screenings were repeated on a yearly basis. Adverse events were assessed clinically at each visit. For outcome assessment, SALT scores were estimated retrospectively based on the patients’ baseline images at initiation and in their last follow-up visit. Time to regrowth was defined as the interval from treatment initiation to the first clinically documented regrowth of terminal hair over previously affected scalp regions. Descriptive statistics were used to summarize the baseline characteristics and treatment outcomes. Continuous variables are presented as medians with ranges, whereas categorical variables are presented as counts.

## 3. Results

Eight pediatric patients with severe alopecia areata were included (Table 1). The median age was 12 years (range: 9–14), and the median disease duration was 7.5 years (range: 2–13). Five patients had alopecia subtotalis, two had alopecia totalis, and one had alopecia universalis. All patients received prior topical therapies, with three patients receiving prior JAK inhibitor treatments. The median time to visible regrowth was 3 months (range: 2–6), and the median follow-up duration was 14.5 months (range: 4–24). Among the five patients with available baseline photographic documentation, the median baseline SALT score was 90 (range: 45–100), which improved to 10 (range: 4–60) at the most recent follow-up visit. One patient experienced treatment interruption, and one patient required dose escalation to 30 mg, alternating with 15 mg. Mild acne was documented as a side effect in two patients; otherwise, no serious adverse events were documented.

### 3.1. Case Presentation

#### 3.1.1. Case 1

**Figure 1:**
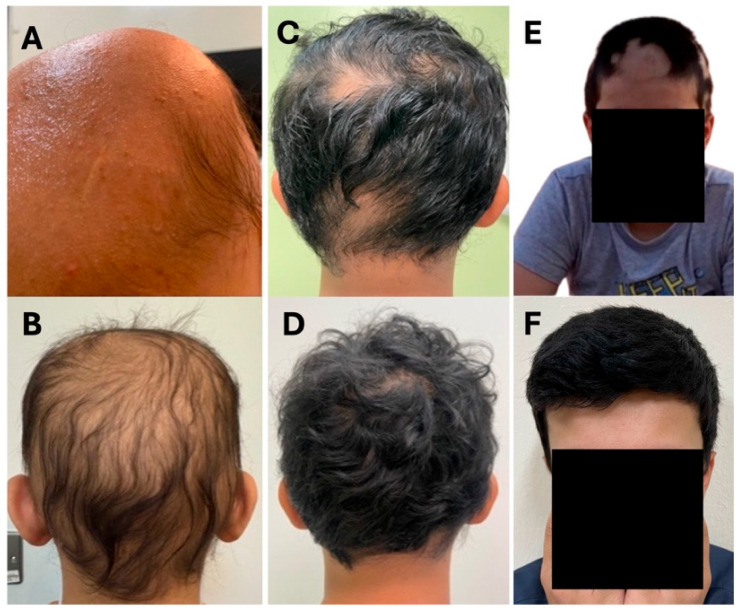
Clinical photographs of patients (Cases 1–3) before and after treatment with upadacitinib. (**A**): Case 1 pre-treatment; (**B**): Case 1 post-treatment; (**C**): Case 2 pre-treatment; (**D**): Case 2 post-treatment; (**E**): Case 3 pre-treatment; (**F**): Case 3 post-treatment.

A 14-year-old female with a long-standing 13-year history of alopecia areata, progressing to alopecia totalis, presented after failing multiple treatments. She had previously received topical therapies, including mometasone furoate and anthralin, with minimal improvement over one year. This was followed by a five-month course of tofacitinib and an 18-month trial of ruxolitinib, both yielding unsatisfactory results (Figure 1A). Upadacitinib was then initiated at 15 mg once daily. After 17 months of treatment, the patient achieved complete regrowth of scalp, eyebrow, and eyelash hair, though with overall low hair density (Figure 1B). At her most recent visit, 3 months after her last one, no more clinical improvement was noted. Thus, dose escalation was initiated, alternating between 15 mg and 30 mg of upadacitinib. The treatment was well-tolerated with no reported side effects or laboratory abnormalities throughout the course. Her estimated SALT score improved from 100 at her initial visit to 60 at the 17-month follow-up visit.

#### 3.1.2. Case 2

A 9-year-old male with an 8-year history of alopecia areata presented with multiple scalp patches and complete loss of eyebrows and eyelashes. He had previously received various topical and systemic treatments, including minoxidil, azathioprine, and systemic steroids, with minimal response. At our clinic, he underwent a 20-month course of multiple topical agents without significant improvement (Figure 1C). Due to lack of efficacy, upadacitinib 15 mg once daily was initiated. After 14 months, he showed near-complete scalp regrowth, with only residual ophiasis pattern hair loss and an estimated SALT score of 10 compared with 45 at presentation (Figure 1D). At 20 months, he achieved full, dense regrowth of scalp, eyebrows, and eyelashes. Three months later, a mild relapse with three new patches was noted, likely due to poor compliance. Throughout treatment, the medication was well-tolerated with no side effects or laboratory abnormalities reported.

#### 3.1.3. Case 3

A 14-year-old male with an 8-year history of alopecia areata, along with comorbid atopic dermatitis and subclinical hypothyroidism, presented with hair loss involving the scalp, eyebrows, and eyelashes. He had been treated with topical agents, including mometasone furoate and minoxidil, for seven years, with minimal to partial improvement (Figure 1E). Upadacitinib was initiated at 15 mg once daily, resulting in near-complete regrowth of scalp, eyebrow, and eyelash hair after four months, with only a small patch over the left eyebrow remaining. After 24 months of continued treatment, the patient maintained his response, with only a small, hidden patch on the occiput noted on exam (Figure 1F). The medication was well-tolerated with no significant side effects except for mild acne, which was successfully managed with topical therapy.

#### 3.1.4. Case 4

**Figure 2:**
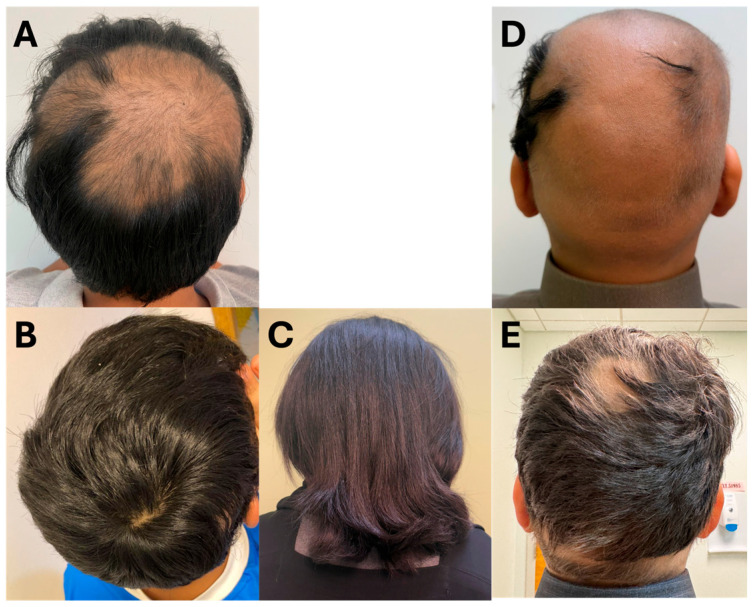
Clinical photographs of patients (Cases 4–6) before and after treatment with upadacitinib. (**A**): Case 4 pre-treatment; (**B**): Case 4 post-treatment; (**C**): Case 5 post-treatment; (**D**): Case 6 pre-treatment; (**E**): Case 6 post-treatment.

An 11-year-old male with a 7-year history of alopecia areata presented with large alopecic patches involving the frontal, parietal, and occipital scalp, which later progressed to near-total scalp and eyebrow involvement with an estimated SALT score of 60. He had previously been treated with topical agents, including mometasone furoate, minoxidil, and clobetasol, with limited benefit. A trial of ruxolitinib was initiated but discontinued due to hyperkalemia and an abnormal liver profile; even after restarting, the response remained poor (Figure 2A). He was then switched to upadacitinib 15 mg once daily, which led to significant improvement within three months, including eyebrow regrowth and cessation of hair fall. Treatment was briefly interrupted due to neutropenia, later attributed to a transient infection and not the medication. After 12 additional months of therapy, the patient achieved near-total scalp regrowth with only two small residual patches and complete eyebrow and eyelash regrowth with an estimated SALT score of 4 (Figure 2B). The medication was well-tolerated with no side effects or lab abnormalities for the remainder of the treatment period.

#### 3.1.5. Case 5

A 12-year-old female with a long-standing history of alopecia areata since the age of 4 progressed from patchy scalp and eyelash involvement to alopecia totalis, including complete loss of scalp, eyebrow, and eyelash hair. She failed to respond to multiple topical treatments as well as two systemic JAK inhibitors—tofacitinib and ruxolitinib—despite extended use. Upadacitinib was initiated at a dose of 15 mg once daily, resulting in visible improvement after 4 months, including regrowth of scalp, eyebrow, and eyelash hair with greater density than previous treatments. By 7 months, only two small occipital patches remained, and at her 12-month follow-up, she achieved complete, dense regrowth of scalp hair along with full and sustained regrowth of eyebrows and eyelashes (Figure 2C). The treatment was well-tolerated, with no reported side effects, lab abnormalities, or complications.

#### 3.1.6. Case 6

A 12-year-old male with a 2-year history of alopecia universalis and comorbid bronchial asthma presented after failing previous treatments, including minoxidil and intralesional triamcinolone, with minimal response and an estimated SALT score of 95 (Figure 2D). He was started on upadacitinib 15 mg once daily and achieved complete regrowth of scalp, eyebrow, and eyelash hair within 6 months. However, after being lost to follow-up and discontinuing the medication, he experienced a relapse of hair loss. Upon re-initiation of upadacitinib at the same dose, vellus hair regrowth on the scalp and full regrowth of eyebrows and eyelashes were noted within 3 months. At his 6-month follow-up, increased growth of dark terminal hair over the scalp was observed. At his 13-month follow-up, the patient showed near-complete scalp hair regrowth with an estimated SALT score of 10 (Figure 2E). The treatment was well-tolerated during both courses, with no reported side effects, lab abnormalities, or complications.

#### 3.1.7. Case 7

**Figure 3:**
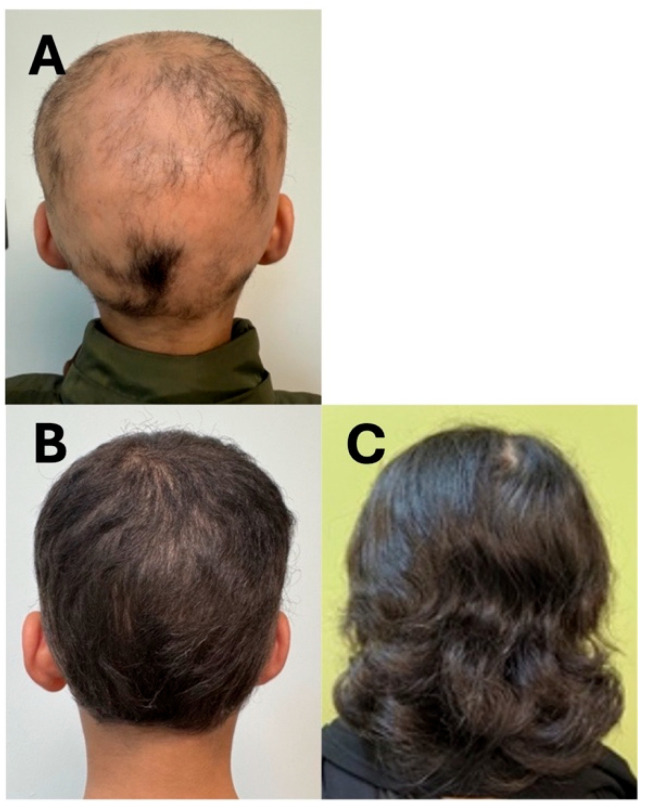
Clinical photographs of patients (Cases 7 and 8) before and after treatment with upadacitinib. (**A**): Case 7 pre-treatment; (**B**): Case 7 post-treatment; (**C**): Case 8 post-treatment.

A 12-year-old male, with a 5-year history of alopecia subtotalis, presented with multiple, nonscarring alopecic patches involving mostly his scalp and, to a lesser extent, his eyebrows (Figure 3A). He had been previously treated with betamethasone cream and minoxidil by other providers; however, he did not demonstrate any clinical improvement. Given his history of failing topical treatments, we started our patient on upadacitinib 15 mg once daily. Four months after starting treatment, the patient demonstrated improvement with complete, dense hair regrowth over the scalp and eyebrows except for a few areas over the right temporal scalp, where low-density hair was noted (Figure 3B). At his initial visit, the estimated SALT score was 90, which improved to 10 at his subsequent visit.

#### 3.1.8. Case 8

A 14-year-old female with a 5-year history of alopecia areata presented with diffuse patchy scalp hair loss, which had progressed to include the eyebrows and eyelashes following an initial response to topical treatments. Upon recurrence, she was started on upadacitinib (15 mg) once daily. Within 3 months, she showed regrowth of scalp, eyebrow, and eyelash hair, with complete regrowth maintained over a 14-month follow-up period (Figure 3C). The treatment was well-tolerated, with no lab abnormalities or significant side effects, except for mild acne, which was successfully managed with topical therapy.

## 4. Discussion

This is one of the largest case series to date that describes the clinical response and safety profile of upadacitinib in the treatment of severe alopecia areata in the adolescent and pediatric age groups. Eight patients between the ages of 9 and 14 were included. The study population demonstrated extended and severe disease, with a median duration of 7.5 years. Five patients exhibited alopecia subtotalis, two exhibited alopecia totalis, and one exhibited alopecia universalis, with several showing involvement beyond the scalp. Three patients had previously undergone treatment with alternative JAK inhibitors, signifying treatment resistance. All of them were treated with upadacitinib at a dose of 15 mg, taken once daily, and all of them demonstrated clinical improvement with minimal to no side effects.

Upadacitinib is a selective JAK1 (Janus kinase 1) inhibitor that is FDA approved for the treatment of autoinflammatory conditions such as rheumatoid arthritis and ankylosing spondylitis in adults. In pediatric patients aged 12 and above, it is FDA approved for the treatment of atopic dermatitis. Most of the existing literature regarding the use of upadacitinib in alopecia areata is limited to the adult patient population, with doses ranging from 15 mg once daily to 30 mg once daily [7,8]. In pediatric patients aged less than 18, lower doses such as 15 mg taken once daily have been used in several cases with demonstrated clinical improvement [9,10]. This cohort is similar to our patients, where a dose of 15 mg taken once daily resulted in hair regrowth as early as 3 months into the medication course.

These observations indicate that clinical improvement can be achieved in this age group with the 15 mg dosage; however, controlled studies are needed to establish the optimal dosing regimen.

The current data discussing JAK inhibitor use in pediatric patients are limited. Most of the available literature discussing agents like tofacitinib, ruxolitinib, and upadacitinib and their role in refractory alopecia areata is confined to case reports. Our case series discusses the use of upadacitinib in different alopecia subtypes and over a longer follow-up compared to previous reports. Our reported findings do not claim superiority of upadacitinib over other JAK inhibitors but rather describe its associated clinical responses in patients with severe alopecia areata [11,12,13].

Regarding the safety and side effect profile, the literature reports acne as an expected side effect of treatment with upadacitinib [14,15,16]. This is consistent with our findings, where two of our patients developed acne; however, we cannot be sure if upadacitinib is involved as acne commonly occurs during the adolescent period. Another side effect experienced by one of our patients was neutropenia, which is similar to one case in the literature mentioning transient mild leukopenia as a side effect of the medication [10]. However, our patient was referred to hematology, and the neutropenia was attributed to an acute viral infection rather than a manifestation of the drug. The laboratory profile of all our patients was normal and displayed no significant elevation in lipid profile throughout the treatment period.

Some of our patients previously used other JAK inhibitors such as tofacitinib and ruxolitinib before being switched to upadacitinib due to suboptimal results. Transitioning to upadacitinib was associated with clinical improvement occurring as early as 3 months, with complete dense hair regrowth after 12–24 months of treatment. These findings indicate a possible therapeutic role in the treatment of alopecia areata in certain patients, but more data and research are needed to confirm this suggestion. Upadacitinib was associated with clinical improvement in different alopecia areata subtypes. Patients with more extensive disease (totalis and universalis) required longer periods of treatment before clinical improvement was seen and demonstrated variable regrowth patterns.

The dose was escalated in one patient to 30 mg. Despite escalation, no further clinical improvement was observed. In another patient, a treatment interruption resulted in relapse; however, clinical improvement was noted upon resumption of therapy. These observations underscore the variability in individual responses, emphasize the necessity of treatment continuity and long-term monitoring, and indicate the imperative for additional research to more precisely delineate predictors of therapeutic response.

This study has several limitations. First, the data were extracted from electronic medical records and included both retrospective and prospective elements. This approach might have introduced documentation bias or inconsistencies. Second, the absence of a control group limits a direct comparison of upadacitinib with other treatment modalities or placebo and restricts the ability to draw definitive conclusions regarding its efficacy in alopecia areata, and the small sample size further limits generalizability of the findings. Third, patients had diverse treatment histories, which included topical treatments and systemic therapies, such as other JAK inhibitors. Although this indicates treatment resistance, prior medication exposure may serve as a confounding variable, obscuring the evaluation of upadacitinib’s independent effect. A further limitation is the incomplete photographic documentation—two patients lacked baseline images prior to starting upadacitinib, hindering full visual comparison of treatment outcomes. Although clinical records were complete, the lack of baseline standardized imaging in these patients reduces the ability to objectively assess initial disease severity and quantify treatment response, which might introduce potential bias in outcome evaluation. Furthermore, objective scoring systems like the SALT (Severity of Alopecia Tool) were estimated retrospectively using available baseline and follow-up photographs. Three patients lacked baseline images; thus, we were unable to precisely estimate their SALT scores. This retrospective estimation may introduce measurement bias and limits the precision of severity assessment. Treatment response was also evaluated qualitatively via clinical assessment, thereby introducing potential subjective bias. Pediatric-specific pharmacokinetics were not evaluated, and all patients received the same dose of 15 mg daily despite varying ages (9–14). Therefore, conclusions about optimal age- or weight-adjusted dosing in younger children could not be drawn.

While the short- and mid-term outcomes were favorable, long-term safety, durability of response, and relapse risk after discontinuation remain uncertain. One patient experienced relapse after stopping treatment, which illustrates the importance of continued monitoring with structured and extended longitudinal follow-up. Based on these preliminary findings, upadacitinib may represent a potential treatment option for refractory pediatric alopecia areata. Future larger-scale research is warranted to validate our findings and explore the therapeutic role of upadacitinib in alopecia areata. The pharmacokinetics of upadacitinib should also be explored to determine the optimal dosing of the drug in the pediatric age group.

## 5. Conclusions

To date, this is one of the largest case series evaluating the efficacy of upadacitinib in pediatric and adolescent patients. All eight of our patients tolerated the drug well and showed encouraging clinical responses to upadacitinib, with a generally benign course of treatment regarding side effects. Some of our patients improved on upadacitinib after experiencing response failures to other JAK inhibitors. Although short-term and mid-term responses were favorable, long-term safety and relapse risk are still unclear, necessitating a larger, controlled study to explore these aspects and validate our study’s findings.

## Figures and Tables

**Table 1:** Baseline characteristics and outcomes of pediatric patients treated with upadacitinib.

Case No.	1	2	3	4	5	6	7	8
Age (yr)	14	9	14	11	12	12	12	14
Sex	F	M	M	M	F	M	M	F
Disease Duration (yrs)	13	8	8	7	8	2	5	5
AA Subtype	Totalis	Subtotalis	Subtotalis	Subtotalis	Totalis	Universalis	Subtotalis	Subtotalis
Baseline SALT	100	45	N/A	60	N/A	95	90	N/A
Follow-up SALT	60	10	N/A	4	N/A	10	10	N/A
Extra-Scalp Involvement	EB, EL	EB, EL	EB, EL	EB, EL	EB, EL	EB, EL	EB	EB, EL
Prior JAK Inhibitor Exposure	Yes	No	No	Yes	Yes	No	No	No
Time to Regrowth (mo)	3	2	3	3	4	6	4	3
Duration of Follow-Up	17	20	24	15	12	13	4	14
Dose (Daily)	15 mg → 15/30 mg alternating	15 mg	15 mg	15 mg	15 mg	15 mg	15 mg	15 mg
Interruption	No	No	No	No	No	Yes	No	No
Adverse Events	No	No	Acne	No	No	No	No	Acne

Abbreviations: F: female; M: male; AA: Alopecia areata; N/A: not applicable; EB, eyebrows; EL, eyelashes; SALT, Severity of Alopecia Tool; JAK, Janus kinase.

## Data Availability

The data presented in this study are available from the corresponding author upon reasonable request. The data were obtained from the BESTCare electronic medical record system at King Abdulaziz Medical City and are not publicly available due to institutional policies and patient confidentiality considerations.

## Abbreviations

The following abbreviations are used in this manuscript:

JAK	Janus kinase
AA	Alopecia areata
IL	interleukin
JAK-STAT	Janus kinase–signal transducer and activator of transcription
FDA	Food and Drug Administration
JAK1	Janus kinase 1
SALT	Severity of Alopecia Tool

## References

[1] Juárez-Rendón K.J., Rivera Sánchez G., Reyes-López M.Á., García-Ortiz J.E., Bocanegra-García V., Guardiola-Avila I., Altamirano-García M.L. (2017). Alopecia areata. Current situation and perspectives. Arch. Argent. Pediatr..

[2] Dainichi T., Iwata M., Kaku Y. (2023). Alopecia areata: What’s new in the epidemiology, comorbidities, and pathogenesis?. J. Dermatol. Sci..

[3] Rajabi F., Drake L.A., Senna M.M., Rezaei N. (2018). Alopecia areata: A review of disease pathogenesis. Br. J. Dermatol..

[4] Barton V.R., Toussi A., Awasthi S., Kiuru M. (2022). Treatment of pediatric alopecia areata: A systematic review. J. Am. Acad. Dermatol..

[5] King B.A., Craiglow B.G. (2023). Janus kinase inhibitors for alopecia areata. J. Am. Acad. Dermatol..

[6] Hamilton C.E., Craiglow B.G. (2020). JAK inhibitors for the treatment of pediatric alopecia areata. J. Investig. Dermatol. Symp. Proc..

[7] Salvi I., Parodi A., Cozzani E., Burlando M. (2024). Psoriasiform eczema with immune-mediated comorbidities treated with upadacitinib: A case report. Front. Immunol..

[8] He X., Yang D., Lai L., Lang J., Wei K., Xiao M. (2024). Upadacitinib for alopecia areata in different backgrounds: A case series. Clin. Cosmet. Investig. Dermatol..

[9] Yu D., Ren Y. (2023). Upadacitinib for successful treatment of alopecia universalis in a child: A case report and literature review. Acta Derm. Venereol..

[10] Kołcz K., Żychowska M., Sawińska E., Reich A. (2023). Alopecia universalis in an adolescent successfully treated with upadacitinib: A case report and review of the literature on the use of JAK inhibitors in pediatric alopecia areata. Dermatol. Ther..

[11] Ma Y., Wang W., Shi D. (2024). Tofacitinib treatment in a severe pediatric alopecia areata: A case report and a literature review. Skin. Res. Technol..

[12] Peterson D.M., Vesely M.D. (2020). Successful treatment of alopecia totalis with ruxolitinib in a preadolescent patient. JAAD Case Rep..

[13] Ha G.U., Kim J.H., Jang Y.H. (2023). Improvement of severe alopecia areata in an adolescent patient on upadacitinib. Pediatr. Dermatol..

[14] Giavina-Bianchi M., Giavina-Bianchi P. (2024). Successful treatment of severe atopic dermatitis and alopecia universalis with upadacitinib in a 29-year-old male patient. J. Allergy Clin. Immunol. Glob..

[15] Johnston L.A., Poelman S.M. (2023). Upadacitinib for Management of Recalcitrant Alopecia Areata: A Retrospective Case Series. JAAD Case Rep..

[16] Novielli D., Foti C., Principi M., Mortato E., Romita P., Dell’AQuila P., Di Leo A., Ambrogio F. (2023). Upadacitinib in concurrent Crohn’s disease, atopic dermatitis and alopecia areata: A case report. J. Eur. Acad. Dermatol. Venereol..

